# Identification of *ZOUPI* Orthologs in Soybean Potentially Involved in Endosperm Breakdown and Embryogenic Development

**DOI:** 10.3389/fpls.2017.00139

**Published:** 2017-02-08

**Authors:** Yaohua Zhang, Xin Li, Suxin Yang, Xianzhong Feng

**Affiliations:** Key Laboratory of Soybean Molecular Design Breeding, Northeast Institute of Geography and Agroecology, Chinese Academy of SciencesChangchun, China

**Keywords:** seed development, endosperm breakdown, transcription factor, *GmZOU*, soybean

## Abstract

Soybean (*Glycine max* Merr.) is the world’s most widely grown legume and provides an important source of protein and oil. Improvement of seed quality requires deep insights into the genetic regulation of seed development. The endosperm serves as a temporary source of nutrients that are transported from maternal to filial tissues, and it also generates signals for proper embryo formation. Endosperm cell death is associated with the processes of nutrient transfer and embryo expansion. The bHLH domain transcription factor AtZHOUPI (AtZOU) plays a key role in both the lysis of the transient endosperm and the formation of embryo cuticle in *Arabidopsis thaliana*. There are two copies of soybean *GmZOU* (*GmZOU-1* and *GmZOU-2*), which fall into the same phylogenetic clade as *AtZOU*. These two copies share the same transcription orientation and are the result of tandem duplication. The expression of *GmZOUs* is limited to the endosperm, where it peaks during the heart embryo stage. When the exogenous *GmZOU-1* and *GmZOU-2* were expressed in the *zou-4* mutant of *Arabidopsis*, only *GmZOU-1* partially complemented the *zou* mutant phenotype, as indicated by endosperm breakdown and embryo cuticle formation in the transgenic lines. This research confirmed that the *GmZOU-1* is a *ZOU* ortholog that may be responsible for endosperm breakdown and embryo cuticle formation in soybean.

## Introduction

Soybean is a major global economic crop and a source of carbohydrates, protein, oil, and other nutrients for humans and animals (Hou et al., 2009). Improvements of the production and seed quality of soybean require a thorough understanding of the underlying processes governing seed growth and development. However, most of the research performed on soybean have focused on various biochemical pathways for producing seed storage products based on known enzymatic steps, and less attention has been given on the relationship between seed development and storage product metabolic programs, e.g., coordination of different seed compartments pertaining to resource channeling and storage product accumulation, and as such, the regulatory and dynamic aspects of the network are still poorly understood.

In dicotyledonous species, the endosperm plays important roles in metabolite production, transport, and accumulation in the embryo (Melkus et al., 2009). The endosperm in Brassicaceae is believed to participate in the flux control of nutrients delivered by the vascular tissues of the parental plants to the embryo (Abbadi and Leckband, 2011). The soluble metabolites are temporarily stored in the endosperm vacuole with the concentration developmentally controlled, and influence metabolite accumulation in embryo (Melkus et al., 2009). The endosperm is also involved in lipid, sugar, amino acid, and organic acid metabolism (Schwender and Ohlrogge, 2002; Hill et al., 2003), and in mineral acquisition and storage (Otegui et al., 2002). In addition to nutrient transport and metabolism, the endosperm also plays a role in controlling the sizes of the embryo and seed, although the precise mechanism is still unknown (Luo et al., 2005).

Endosperm development in most angiosperms is of the nuclear type and is characterized by four phases: syncytial, cellularization, differentiation, and death (Olsen, 2004). After cellularization, the embryo surrounding region (ESR) of the endosperm cell starts to breakdown, thereby freeing the nutrients that fuel the embryo and in turn create space for embryo expansion (Ingram, 2010). In dicotyledons such as *Arabidopsis* and soybean, the endosperm undergoes programmed cell death with complete autolysis, leaving a single cell layer of endosperm tissue surrounding the dormant embryo (Ingram, 2010). The endosperm death stage, which represents the beginning of the maturation stage, refers to a process involving nutrient transfer and storage in the embryo, as well as the generation of developmental signals that are transmitted to the young embryo (Berger, 2003; Sharma et al., 2003; Berger et al., 2006; Baud et al., 2008). During this stage, the sucrose transporter *AtSUC5* is induced in the ESR region and plays an important yet transient role in the transport of nutrients to the embryo (Baud et al., 2005). In pea and *Vicia* spp. seeds, a change from a predominantly hexose to sucrose content in the endosperm induces a change of gene expression in favor of storage product accumulation (Hill et al., 2003). *SBT1.1*, a subtilase gene, is specifically expressed in the endosperm of *Medicago truncatula* and *Pisum sativum* seeds to control embryo growth (D’Erfurth et al., 2012). The endosperm degradation stage is a transition process that influences seed development and metabolite accumulation.

The *ZOUPI* (*ZOU*) gene is a unique and highly conserved bHLH transcription factor that is exclusively expressed in the ESR and controls both endosperm breakdown and embryonic cuticle formation during *Arabidopsis* seed development (Kondou et al., 2008; Yang et al., 2008). In *zou*/*rge1* mutants, ESR cell death is compromised, and the endosperm persists in the mature seeds with heart shaped embryo. In addition the defect in the embryonic cuticle of *zou* mutants lead to splits in the cotyledon epidermis after germination. The *ZOU* gene is also specifically expressed in the maize endosperm, influencing ESR breakdown and embryonic and seed development (Grimault et al., 2015). The conserved function of the *ZOU* gene in both *Arabidopsis* and maize indicates that it plays an important role in the communication between endosperm breakdown and embryo development.

In order to investigate the regulating pathway of endosperm breakdown and the communication between endosperm and embryo in soybean, we identified a *ZOU* ortholog gene, which complements the *Arabidopsis zou* mutant phenotype allowing the recovery of endosperm breakdown and embryo cuticle formation.

## Materials and Methods

### Plant Materials and Growth Conditions

Soybean plants [*G. max* (L.) Merrill] (Williams *82*) were grown under natural conditions in the field at the Agricultural Experiment Station of Northeast Institute of Geography and Agroecology, Chinese Academy of Sciences, Changchun, China. The *Arabidopsis zou* mutant *zou-4* (Col-0 background) allele was obtained from the Gabi-Kat collection of T-DNA inserts (Rosso et al., 2003) and corresponded to GABI_584D09. *Arabidopsis* seeds were surface-sterilized, vernalized at 4°C for 3 days, and then allowed to germinate and grow on plant growth medium in the growth chamber maintained under a 16-h light/8-h dark (22–18°C) photoperiod. Soil-grown plants were also maintained under the same conditions.

### *GmZOU* Cloning by RT-PCR

Total RNA was isolated from soybean developing seeds under normal growth conditions using a Plant Total RNA Extraction Kit (Bioteke Corporation) and first strand cDNA was prepared from 0.5 μg of total RNA primed with an oligo-dT primer using an AMV reverse transcription system (Takara) according to the manufacturer’s instructions. The genes were cloned using PrimeSTAR HS DNA Polymerase (Takara). Two PCR primers, OL0085 and OL0086, were designed to amplify the *GmZOU-1* gene. A 937-bp PCR fragment was amplified, and the PCR reactions were performed as follows: 98°C for 3 min and then 30 cycles of 98°C for 10 s, 55°C for 15 s, and 72°C for 1 min. Primers OL0087 and OL0088 were designed to amplify the *GmZOU-2* gene. A 830-bp PCR fragment was amplified and the PCR reactions were performed as follows: 98°C for 3 min and then 30 cycles of 98°C for 10 s, 55°C for 15 s, and 72°C for 50 s. 3′ A overhangs were attached to the PCR fragments by using EasyTaq (TransGenBiotech) before their cloning into a pMD18-T vector (Takara).

### Phylogenetic Analysis

The ZOU protein sequences from different species with similarities to AtZOU were mined from Phytozome V11^1^ and GenBank (National Institutes of Health genetic sequence database). The amino acid sequences were aligned using CLUSTALW (Thompson et al., 1994). We limited the boundaries of the sequences. In total, 21 sequences (**Supplementary Dataset 1**) were analyzed. Phylogenetic reconstruction of the *ZOU* genes was performed using the neighbor-joining (NJ) method (Saitou and Nei, 1987). The bootstrap consensus tree inferred from 1,000 replicates was used to represent the evolutionary history of the analyzed taxa (Felsenstein, 1985). Branches corresponding to partitions reproduced in less than 50% bootstrap replicates were collapsed. The percentage of replicate trees in which the associated taxa were clustered together in the bootstrap test (1,000 replicates) was shown next to the branches (Felsenstein, 1985). The tree was drawn to scale, with branch lengths in the same units as those of the evolutionary distances that were used to infer the phylogenetic tree. The evolutionary distances were computed using the Poisson correction method and are in the units of the number of amino acid substitutions per site. All positions containing alignment gaps and missing data were eliminated only in pairwise sequence comparisons (pairwise deletion option). A total of 451 positions were included in the final dataset. Phylogenetic analyses were conducted using MEGA4 (Tamura et al., 2007).

### Quantitative Gene Expression Analysis

In soybean, total RNA was extracted from different tissues, including root, stem, leaf, inflorescence, unopened flower, opened flower, and seeds at 5 DAP (globular stage), 9 DAP (early heart stage), 12 DAP (late heart stage), and 16 DAP (cotyledon stage; Jones and Vodkin, 2013). To obtain the endosperm and embryo separately from soybean seeds, the seeds were dissected transversely (slightly out of the middle toward the micropylar end) using forceps and a microtome blade (Leica). The embryo was picked out by forceps and the endosperm was separated with integument using a scalpel knife under the stereo microscope (Olympus SZX7), and material was flash frozen in liquid nitrogen. In *Arabidopsis*, total RNA was extracted from siliques with seeds developing to the heart stage (about 5 DAP). The total RNA was treated with DNase I (Takara) according to the manufacturer’s instructions. RNA concentrations were determined by using a Nanodrop Spectrophotometer (NanoDrop Technologies). First-strand cDNA was prepared from 0.5 μg of total RNA primed with oligo-dT primer using an AMV reverse transcription system (Takara). PCR reactions were prepared using SYBR Premix EX Taq (Takara) and each 25 μl reaction was triplicated (technical replicates) and for each experiment three biological replicates (i.e., independent plant samples or different plants in the same transgenic line) were made. According to the manufacturer’s protocol, the following program was used: 10 min at 95°C, followed by 40 cycles of 95°C for 10 s and 60°C for 1 min. The products were quantified using a Bio-Rad DNA Engine Opticon 2 real-time PCR machine and an associated software to assay SYBR green fluorescence. Expression levels were calculated using *ACTIN11* (Soybean), which is a commonly used reference gene with stable expression in different tissues (Hu et al., 2009), and *EIF4A1* (*Arabidopsis*) in developing seed as described (Yang et al., 2008; Xing et al., 2013; Grimault et al., 2015). The primers used are listed in **Supplementary Table S1**.

### Vector Construction and Plant Transformation

To construct the *pAtZOU::GmZOU-1* and *pAtZOU::GmZOU-2* expression vectors, cloned *GmZOU-1* and *GmZOU-2* CDS in pMD18-T vectors in the correct orientation were digested with *SalI* and *SmaI* and cloned into the vector pRT101 that was digested by *XhoI* and *HincII*, thereby constructing the vectors ZYH051 and ZYH052. ZYH051 and ZYH052 were then digested with *PstI*, and the gene fragments were separately cloned into a binary vector pCAMBIA1300 to construct the vectors, ZYH053 and ZYH054. A 1.6-kb region upstream from the *AtZOU* start codon was amplified by PCR (as described earlier) using primers ZOUSALIF and ZOUSALIR and subcloned into vector pSC-B (Stratagene). The vector containing *AtZOU* promoter was digested with *SalI*, and the promoter fragment was then cloned into the binary vectors ZYH053 and ZYH054, thereby constructing the *pAtZOU::GmZOU-1* and *pAtZOU::GmZOU-2* expression vectors ZYH055 and ZYH056. All recombinant plasmids identified from individual *Escherichia coli* colonies were verified by sequencing, the expression vectors described above were introduced into *Agrobacterium tumefaciens* GV3101 independently for plant transformation. Plant transformations were conducted according to Clough and Bent (1998). Plants were dipped twice in a solution of 10% sucrose, 0.5 × MS salts, and 0.05% silwet L-77 solution containing GV3101 Agrobacterium cells that were grown for 48 h.

### Selection of Transformed Plants

Seeds from the transformed plants were screened on MS agar (Sigma) containing 2% sucrose, 20 mg L^-1^ hygromycin (Roche) and 100 mg L^-1^ cefotaxime (Sangong). Resistant plants were transferred to pots with soil and perlite (3:1 v/v) and grown to maturity in a growth chamber. Hygromycin-resistant plants were screened for the presence of the transgene by PCR using soybean *GmZOUs*-specific primers (above). Seeds were harvested and sown on MS agar containing 2% sucrose, 20 mg L^-1^ hygromycin to obtain homozygous plants. Homozygous plants were transferred to soil and were grown for seed production. Seeds from these homozygous plants were used in the subsequent experiments.

### Toluidine Blue Staining

The Toluidine Blue (TB) test was carried out following published procedures (Tanaka et al., 2004; Xing et al., 2013). Seeds were spread uniformly on 15-cm plates containing 1 × MS Basal Salts (Sigma), 0.3% sucrose, and 0.4% Phytagel (Sigma; pH 5.8). Stratification was conducted at 4°C for 3 days before transferring plates to a growth chamber for 7 days. Lids were removed and plates were immediately flooded with the staining solution [0.05% (w/v) TB + 0.4% (v/v) Tween-20] for 2 min. The staining solution was poured off, and the plates were immediately rinsed gently by flooding under a running tap until the water cleared (1–2 min). Seedlings were photographed, or harvested for TB quantification. To harvest, seedlings were removed individually from plates and both roots and any adhering seed coats (both of which stain darkly with TB) were completely removed before plunging the hypocotyl and cotyledons into 1 ml of 80% ethanol. Seedlings were incubated with continuous shaking for 2 h, until all blue color and chlorophyll had been removed from cotyledons. The resulting liquid was analyzed using a spectrophotometer.

### Seed Clearing

To visualize and stage the developing seeds, siliques were opened with needles. The seeds were then cleared overnight in modified Hoyer’s solution (chloral hydrate: water: glycerol in proportions 8 g : 2 mL : 1 mL) and visualized under DIC optics using a Nikon Eclipse E600 microscope.

## Results

### *GmZOU* Genes Cloned from Developing Seeds of Soybean

**FIGURE 1:**
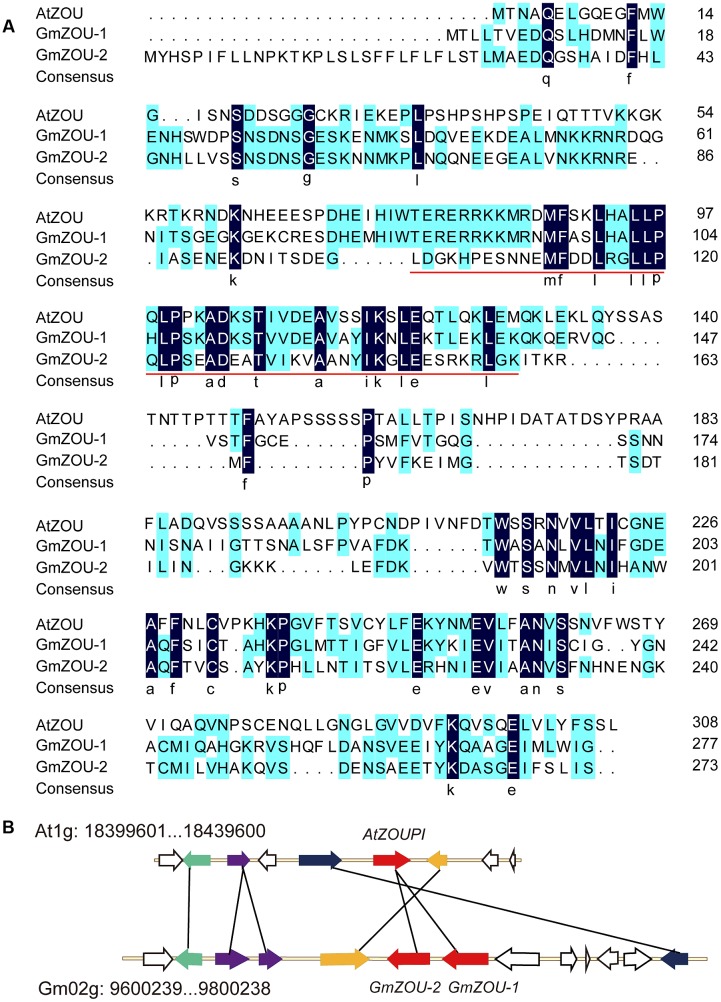
**Identification of *ZOU* homologs in soybean.** **(A)** Alignment of amino acid sequences of the AtZOU gene with GmZOU-1 (Glyma.02G103200) and GmZOU-2 (Glyma.02G103100). Identical amino acids are indicated by white letters against a black background and the conserved bHLH domain (pfam00010) was remarked by red line. **(B)** A syntenic block of soybean and *Arabidopsis thaliana* sequence assemblies surrounding the *Arabidopsis ZOU* gene. Solid black lines connect homolog gene pairs and are indicate by the same color. White arrows represent genes with no syntenic homolog(s) in the particular genomic region. The *ZOUPI* tandem duplicates were detected in soybean (*GmZOU-1* and *GmZOU-2*).

To identify the *ZOU* homologs in soybean, the TBLASTN program was used to query the deduced amino acid sequences of gene models from the soybean genomic sequence database (Phytozome) with that of AtZOU. The two highest scores were obtained for the genes models *Glyma.02G103200* and *Glyma.02G103100*, and the corresponding genes were designated as *GmZOU-1* and *GmZOU-2*, respectively. Alignment of the amino acid sequences of the *GmZOU-1* and *GmZOU-2* with that of *AtZOU* revealed the presence of a basic helix-loop-helix (bHLH) DNA-binding domain (pfam00010) and a conserved C-terminal domain (**Figure 1A**). Phylogenetic analysis of the all 480 bHLH family proteins in soybean and the *AtZOU* protein showed that both *GmZOUs* were in the same phylogenetic clade with *AtZOU* (**Supplementary Figure S1**).

The two copies of *GmZOUs* identified in soybean were located on Gm02: 9,795,987–9,799,196 (Accession Number *Glyma.02G103200*) and Gm02: 9,784,634–9,786,935 (Accession Number *Glyma.02G103100*), which is adjacent to the chromosome with the same transcription orientation. The chromosome fragment in soybean chromosome 2 containing 13 genes around the *GmZOUs* was aligned with the chromosome fragment containing *AtZOU* from the *Arabidopsis* genome. There were five genes in this selected *Arabidopsis* chromosome region that corresponded to seven homologs in this soybean chromosomal segment that contained two genes that were tandemly duplicated. In addition, these homologs showed synteny, as indicated by the same order and orientation (**Figure 1B**). The two chromosomal regions showed similar evolutionary origin, and the two copies of *GmZOUs* were the result of tandem gene duplication.

### Phylogenetic Analysis of *GmZOUs*

**FIGURE 2:**
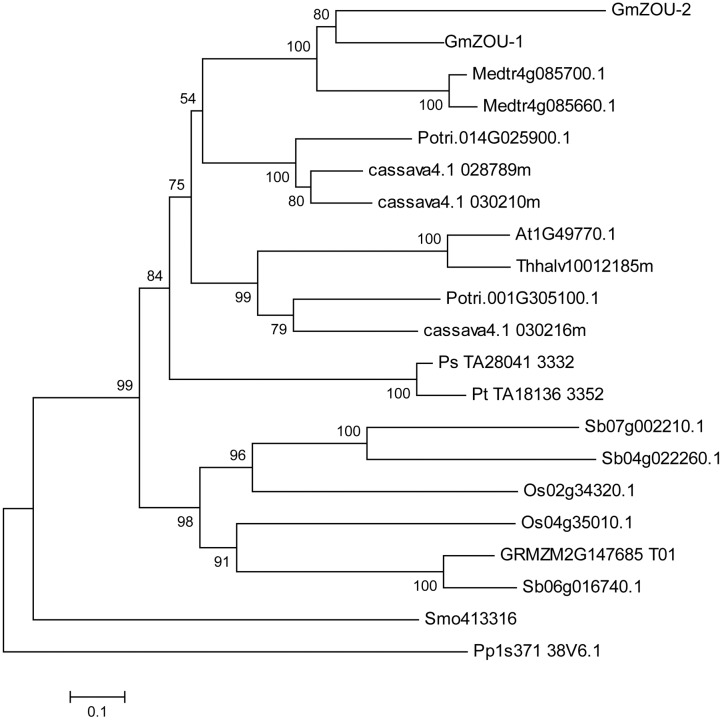
**Phylogenetic analysis of *ZOU.*** A Neighbor-joining tree of *ZOU* homologs from Arabidopsis (*A. thaliana, At*), salt cress (*T. halophila, Thhalv*), soybean (*G. max, Glyma*), lucerne (*M. truncatula, Medtr*), poplar (*P. trichocarpa, Potri*), cassava (*M. esculenta, cassava*), rice (*O. sativa, Os*), corn (*Z. mays, GRMZM*), sorghum (*S. bicolor. Sb*), pine (*P. taeda, Pt*), spruce (*P. sitchensis, Ps*), selaginella (*S. moellendorffii, Smo*), and moss (*P. patens, Pp*). Bootstrapping values are indicated where the local bootstrap probability of each branch is >50.

To identify the phylogenetic relationships and functional conservation of different species during evolution, the homolog of the *ZOU* gene in other species, including *A. thaliana*, salt cress (*Thellungiella halophila*), soybean (*Glycine max*), lucerne (*M. truncatula*), poplar (*Populus trichocarpa*), cassava (*Manihot esculenta*), rice (*Oryza sativa*), corn (*Zea mays*), sorghum (*Sorghum bicolor*), pine (*Pinus taeda*), spruce (*Picea sitchensis*), selaginella (*Selaginella moellendorffii*), and moss (*Physcomitrella patens*), were downloaded and filtered. Only full-length sequences and those with at least 30% similarity with the *AtZOU* gene were included in the analysis. A total of 21 sequences were selected and used in the subsequent NJ phylogenetic analysis. A strict consensus tree was also generated (**Figure 2**).

The phylogenetic tree showed that *ZOU* is widely conserved in plants. Within angiosperms, *ZOU* was found in both plants that develop persistent endosperm and plants that do not as well as in seeded plants lacking endosperm (e.g., *P. sitchensis*, a gymnosperm). It was also found in more basal vascular plant groups, such as the moss (e.g., *P. patens*) and ferns (e.g., *S. moellendorffii*), which lack seeds altogether. Besides that the *ZOU* genes in gymnosperms such as *P. sitchensis* and *P. taeda* had a closer relationship with dicotyledonous species compared to monocotyledonous species. This may be related to the specific function of the *ZOU* gene, which regulated the degeneration of maternal material during gametogenesis. In monocotyledonous species, the gametogenesis evolved in a specific manner wherein the maternally derived nutrients are stored in the endosperm until seed germination. Although ZOU in monocotyledonous species, like AtZOU is involved in the local lysis of endosperm to allow embryo growth, the bulk of persistent endosperm implies some functional alterations. Recently the potential functional specificity of ZmZOU unique to monocotyledon had also been shown in maize (Grimault et al., 2015).

### Expression Profiles of *GmZOUs* in Vegetatives

**FIGURE 3:**
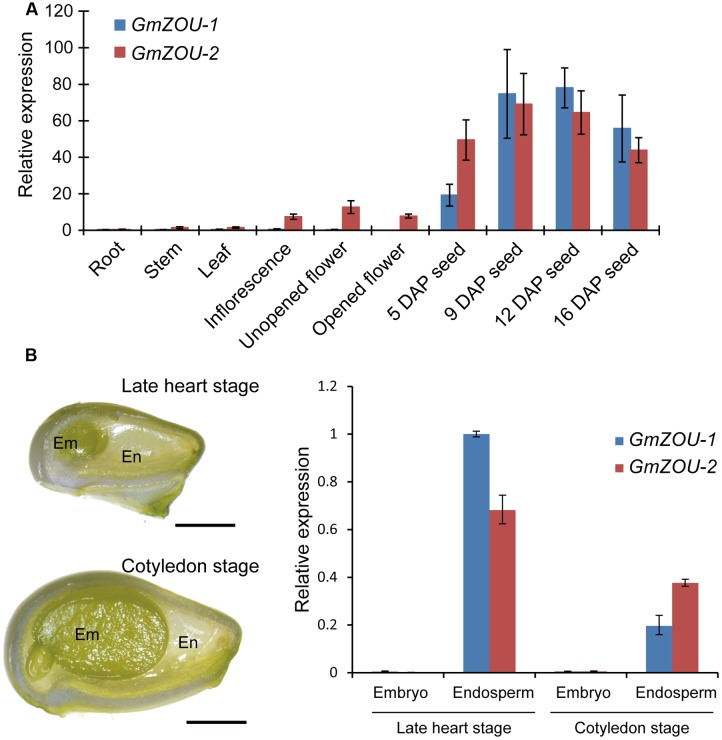
**Expression analyses of *GmZOU-1* and *GmZOU-2* in soybean.** **(A)** The expression of *GmZOUs* in different tissues, including the root, stem, leaf, inflorescence, unopened flower, opened flower, and seeds at different DAP, as detected by qPCR analysis. **(B)** qPCR analysis of *GmZOUs* expression on dissected seed compartments. The embryo and endosperm were separately isolated from developing seeds at late heart stage or cotyledon stage. For each stage at least 20 seeds were selected with three biological replicates. Scale bars: 1 mm.

The expression profiles of the *GmZOUs* in both vegetative and reproductive soybean organs, including roots, stems, leaves, inflorescences, unopened flowers, opened flowers and developing seeds with embryos in different stages were examined by qPCR analysis. The results showed that both *GmZOUs* were highly expressed in developing seeds and preferentially at the heart stage. *GmZOU-2* was also detected in the inflorescences, unopened flowers, and opened flowers (**Figure 3A**).

In order to study the spatial-specific expression pattern of *GmZOUs* in developing seeds, gene expression analysis had been carried out on dissected seed compartments. The embryo and endosperm were separately isolated from developing seeds at late heart stage or cotyledon stage. In order to prevent contamination, the expression of embryo specific genes (*Glyma03g03500* and *Glyma14g35560*) and endosperm specific genes (*Glyma07g02220* and *Glyma08g21890*; Danzer et al., 2015) was also checked in these dissected seed compartments (**Supplementary Figure S2**). Upon verification, *GmZOUs* expression was detected by qPCR analysis. The results showed that both of *GmZOUs* were highly expressed in endosperm whereas no expression was detected in embryo (**Figure 3B**). The similar expression pattern with *AtZOU* implied functional conservation of *GmZOUs* in seed development.

### The Expression of *GmZOUs* in the *Arabidopsis zou* Mutant Facilitates the Recovery of the Mutant Seed Phenotype

**FIGURE 4:**
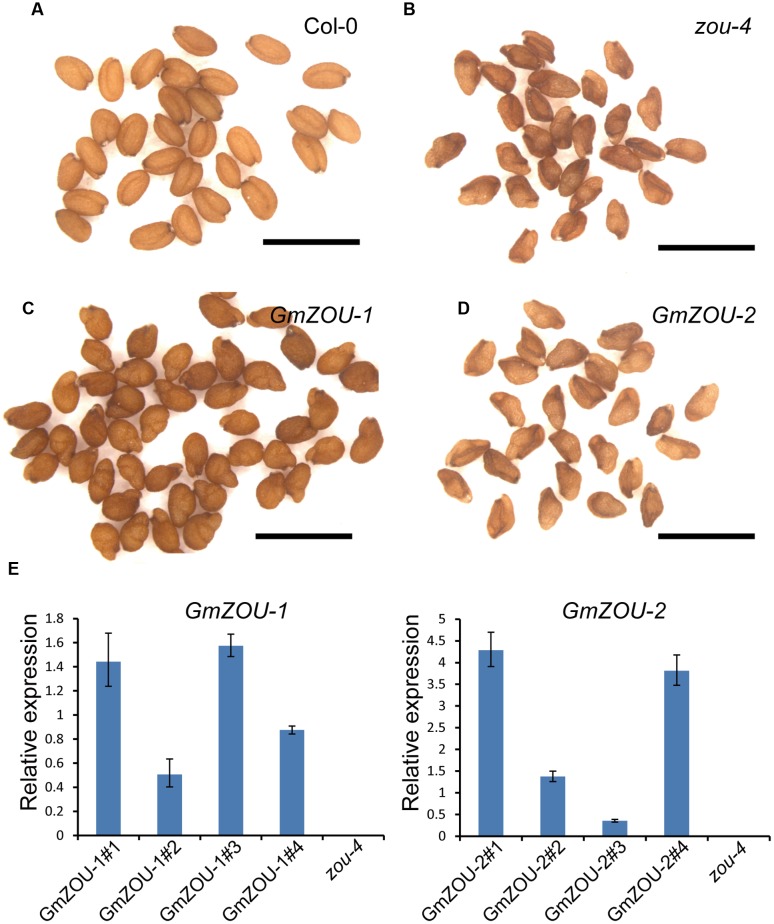
***GmZOUs* expressed in *Arabidopsis zou-4* mutant.** **(A–D)** Seed phenotype of *GmZOU-1* and *GmZOU-2* transgenic lines. The seeds from Col-0 are full, with the embryo showing a perfectly bent shape **(A)** and from *zou-4* are abnormal with a shriveled seed phenotype **(B)**. In *GmZOU-1* transgenic lines, the seeds showed a wild-type-like or malformed seed phenotype **(C)**, whereas the seeds in the *GmZOU-2* transgenic lines showed the shriveled seed phenotype, similar to that in the *Atzou-4* mutant **(D)**. Scale bars: 1 mm. **(E)** qPCR analysis of the *GmZOUs* expression in transgenic lines. Four independent lines from each were selected for examining the exogenous *GmZOUs* expression, which was quantified relative to *AtZOU* expression in Col-0 wild type seeds at the heart stage. The un-transformed *Atzou-4* mutants were selected as a negative control.

To confirm that *GmZOU*s are involved in endosperm breakdown, expression vectors with *GmZOU-1* and *GmZOU-2* under the control of the *AtZOU* promoter (*AtZOUpro-GmZOUs*) were separately transformed into *Atzou-4* mutants. After resistance screening, presence of exogenous *AtZOU* promoter-driven *GmZOU-1* or *GmZOU-2* constructs was checked in the transgenic lines containing the T-DNA insertions. The *Atzou-4* mutant background of the *GmZOU-1* and *GmZOU-2* transgenic lines was then confirmed by PCR analysis based on the T-DNA insertion site. Furthermore, the expression of exogenous *GmZOU*s was also detected by qPCR (**Figure 4E**). Background identification and exogenous *GmZOUs* expression analysis identified a total of four *GmZOU-1* transgenic lines and four *GmZOU-2* transgenic lines, which were then used in the subsequent analysis. Among these transgenic lines, the mature seeds in the *GmZOU-1* transgenic lines rescued the *Atzou-4* shriveled seed phenotype into wild-type-like or malformed seeds (**Figures 4A,C**), whereas the mature seeds in the *GmZOU-2* transgenic lines showed no distinct phenotypic differences from that of *Atzou-4* mutant seeds (**Figures 4B,D**).

**FIGURE 5:**
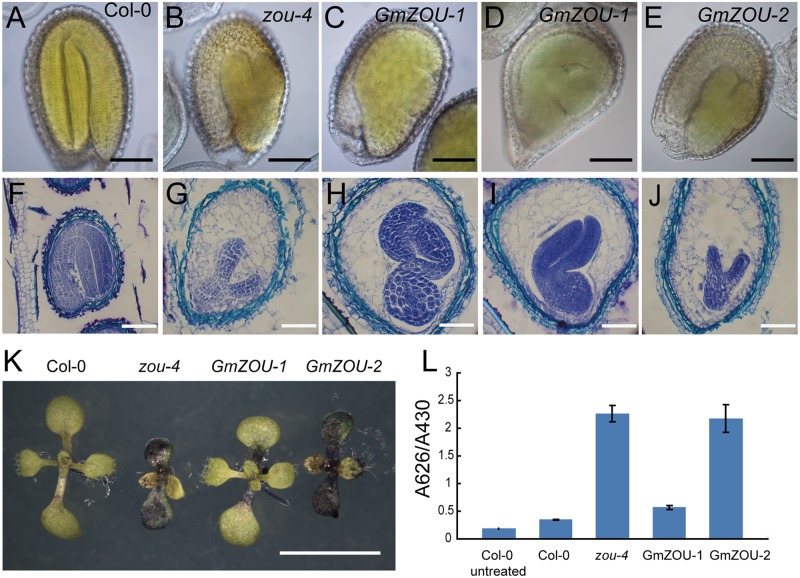
**Evaluation of seed development and detection of the embryo cuticle.** **(A–E)** The seeds at 12 DAP were cleared and observed by DIC microscopy. The embryo in Col-0 showed complete bending, occupying the entire seed cavity **(A)** and arrested in the heart/torpedo stage in the *Atzou-4* mutant **(B)**. In *GmZOU-1* transgenic seeds, the semi-bent and larger embryo occupied the seed **(C,D)**, and the embryo in the *GmZOU-2* transgenic seeds was the same as that in the *Atzou-4* mutant **(E)**. Scale bars: 100 μm. **(F–J)** Embryonic and endosperm development was detected by paraffin sectioning at about 10 DAP. The wild-type seeds were in the mature stage, showing a completely bent embryo and only one cell layer of endosperm surrounding the embryo **(F)**; the *Atzou-4* mutant embryos were arrested in the heart stage and their cellularized endosperm remained intact **(G)**. In *GmZOU-1* transgenic seeds, the endosperm showed breakdown and the embryo expanded to later stages. Furthermore, the expanded embryo still exhibited non-uniform development based on the amount of residual endosperm **(H,I)**. In the *GmZOU-2* transgenic seeds, the embryo and endosperm remained arrested, similar to that in the *Atzou-4* mutant **(J)**. Scale bars: 100 μm in **(F)** and 50 μm in **(G–J)**. **(K,L)** *GmZOU-1* partially recovered the cuticle deficiency in *Atzou-4*. The seedlings of Col-0, *Atzou-4*, and the *GmZOUs* transgenic lines at about 7 days after germination were stained with TB. The *GmZOU-1* transgenic seedlings were weakly stained, whereas the *GmZOU-2* transgenic seedlings showed strong staining similar to that in the *Atzou-4* mutants **(K)**. TB uptake in these lines was quantified spectrophotometrically, showing similar results to that of microscopy analysis. Error bars represent s.d. among three biological replicates, each containing 20 seedlings **(L)**. Scale bars: 5 mm.

To study the embryo and endosperm growth in these transgenic lines, the developing seeds at about 10–12 days after pollination (DAP) were observed. The Col-0 wild-type embryos were in the mature stage showing a completely bent embryo, and the endosperm had already undergone complete breakdown with only one cell layer surrounding the embryo (**Figures 5A,F**); the embryo growth in *Atzou-4* mutant was restricted at the heart/torpedo shaped embryo, and the endosperm failed to undergo a breakdown, with cellularized endosperm persisting (**Figures 5B,G**). The seeds of the *GmZOU-2* transgenic lines showed similar features as that of the *Atzou-4* mutant, with an embryo arrested in the heart/torpedo stage andx showing a sustained cellularized endosperm (**Figures 5E,J**). On the contrary, the embryo of the *GmZOU-1* transgenic lines showed elongated and bending, with the semi-bent embryo almost occupying the entire embryonic sac (**Figures 5C,D**); and the endosperm showed breakdown in *GmZOU-1* transgenic lines resulting in a reduction in the amount of persistent endosperm compared with *Atzou-4* mutant (**Figures 5H,I**). The findings of seed development observation confirmed that the *GmZOU-1* partially recovered embryo expansion and endosperm breakdown in *Atzou-4* mutants, whereas *GmZOU-2* did not influence *Atzou-4* mutant seed development.

Furthermore, we also studied the embryonic cuticle in the transgenic lines as the cuticle integrity in *zou* was not intact with breaks in epidermal. TB, a hydrophilic dye, was used to detect the hydrophobic cuticle and epidermal defection (Tanaka et al., 2004). The seedlings of Col-0, *Atzou-4*, and the *GmZOUs* transgenic lines at about 7 days after germination were stained with TB and visualized under a dissecting microscope. Cotyledons of the *Atzou-4* mutants showed strong staining, while the cotyledons of Col-0 wild-type had no visible permeability to TB. The transgenic *GmZOU-2* cotyledons also showed strong staining similar to that in the *Atzou-4* mutants, whereas the *GmZOU-1* transgenic lines were weakly stained (**Figure 5K**). To further investigate this phenotype, TB uptake by seedlings was quantified by using a spectrophotometer. The results were similar to that of microscopy analysis (**Figure 5L**). These findings indicate that the *GmZOU-1* transgenic plants also partially rescued cuticle formation, whereas *GmZOU-2* did not.

### *GmZOU-1* Recovered the Expression of Presumed ZOU Target Genes

**FIGURE 6:**
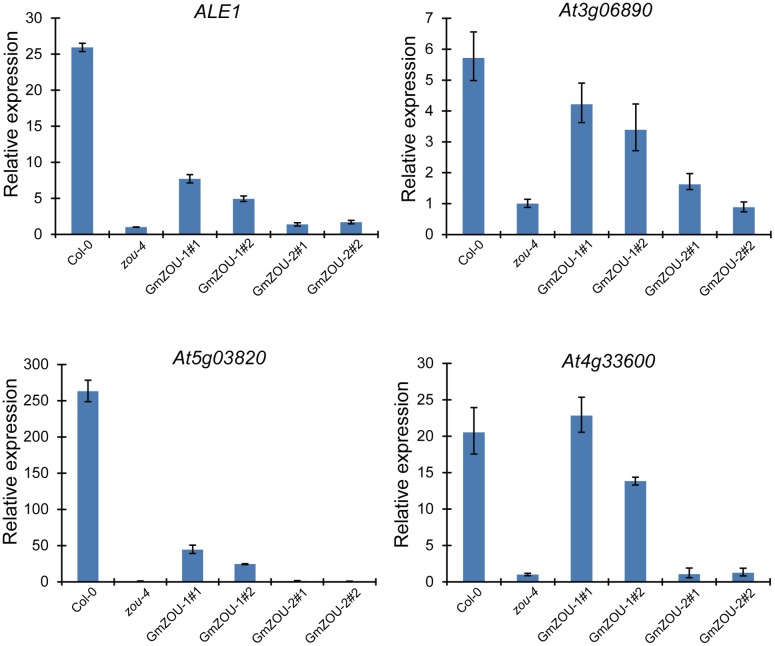
**The expression of AtZOU regulated genes in Col-0, *Atzou-4, GmZOU-1*, and *GmZOU-2* transgenic lines as detected by qPCR.** The expression of *ALE1. At3g06890. At5g03820*, and *At4g33600*, which are the AtZOU presumed target genes, decreased in the *Atzou-4* mutant and showed partial recovery in the *GmZOU-1* transgenic lines, but not in the *GmZOU-2* transgenic lines.

The expression of presumed ZOU target genes, including *ALE1. At3g06890. At5g03820*, and *At4g33600* (Kondou et al., 2008; Yang et al., 2008; Xing et al., 2013) were detected in the *GmZOUs* transgenic lines (**Figure 6**). The ZOU target genes *At3g06890* and *At4g33600* showed almost full recovery in the *GmZOU-1* transgenic lines. The mRNA levels of the putative direct target *ALE1* (Yang et al., 2008; Xing et al., 2013) increased sevenfold and threefold in the *GmZOU-1* transgenic lines compared with levels observed in untransformed *Atzou-4* mutants but reached only 30 and 13%, respectively, of the level observed in Col-0. Similarly, the expression of target gene *At5g03820* in the *GmZOU-1* transgenic lines reached only 17 and 9.3%, respectively, relative to that in Col-0. On the other hand, the expression of these target genes did not differ in the *GmZOU-2* transgenic lines compared to that in the *Atzou-4* mutant.

These results showed that the downregulated genes in the *Atzou-4* mutant underwent partial recovery in the *GmZOU-1* transgenic lines, whereas these did not change in the *GmZOU-2* transgenic lines. In the *GmZOU-1* transgenic lines, the partially recovered expression of these target genes explained the observed partial recovery of the *Atzou-4* mutant phenotype. The less efficient recovery of the ZOU target genes such as *ALE1* and *At5g03820* implied that part of the ZOU target genes requires higher expression levels or complete ZOU function. Nonetheless, our data confirmed that GmZOU-1 is a functional ortholog of AtZOU because both soybean and *Arabidopsis* ZOU induced the upregulation of ZOU presumed target genes, which have been associated with endosperm breakdown and embryo cuticle development.

## Discussion

### *GmZOU-1* Is the *ZOU* Ortholog in Soybean

Searching the soybean genomic sequence database identified two *AtZOU* homologs in soybean, which were then further confirmed by sequence alignment and chromosome synteny analysis. The results also confirmed that the *AtZOU* homologs resulted from tandem duplication. To confirm functional conservation, the *GmZOUs* were expressed in *Arabidopsis Atzou-4* mutants and only *GmZOU-1* could complement the *Atzou-4* mutant phenotype with endosperm breakdown, embryo expansion, and cuticle formation. Additionally, the homologs showed expression patterns that were similar to that of *AtZOU*, which indicated that *GmZOU-1* is the *ZOU* ortholog in soybean. *GmZOU-1* only partially complemented the *Atzou-4* mutant, which was indicative of species specificity or functional diversity during evolution.

### *GmZOU-2* Is Derived from Gene Duplication and Lost Function during the Course of Evolution

*GmZOU-1* (*Glyma.02G103200*) and *GmZOU-2* (*Glyma.02G103100*) resulted from tandem duplication with the same transcription orientation. Sequence alignment indicated that GmZOU-1 consisted of a classical bHLH DNA binding domain at the N terminus and a conserved functional domain at the C terminus compared to AtZOU. By contrast GmZOU-2 only had the conserved function domain, whereas the bHLH domain had lost the conserved amino acids which may influence its DNA binding capability. Transgenic analysis indicated that *GmZOU-2* did not influence the *Atzou-4* mutant in terms of embryonic arrest at the heart stage, sustaining the endosperm, and cuticle formation deficiency. Additionally, considering its less specific expression pattern, we concluded that *GmZOU-2* is the product of gene duplication and may have lost its original function during the course of evolution.

### Cuticle Deficiency and Arrested Endosperm Breakdown Result in Embryonic Malformation in *GmZOU-1* Transgenic Lines

The cuticularization of juxtaposed surfaces has been shown to be extremely important in defining organ boundaries. Mutants with compromised cuticles often show extensive organ fusions (Kurdyukov et al., 2006; Xing et al., 2013). In the *GmZOU-1* transgenic lines, *ALE1* expression was partially recovered, and TB staining showed that the cuticle layer of the embryonic cotyledons did not show complete recovery. The embryo with the defective cuticle readily fused with the endosperm, similar to that observed in other embryo cuticle-deficient mutants such as *ale1. gso1*/*gso2*, and *zou* (Tanaka et al., 2001; Tsuwamoto et al., 2008; Yang et al., 2008). We inferred that the observed organ fusion suppressed embryo expansion. The combination of organ fusion in the embryo and the arrest of endosperm degradation restricted embryonic development, which resulted in the reverse bending of the embryo and a severe malformation phenotype (**Figure 5**). These mechanisms may explain the development of malformed embryos in the *GmZOU-1* transgenic seeds.

## Author Contributions

SY and XF conceived the project and designed this work. YZ cloned the *GmZOUs* and performed phylogenetic and gene expression analyses. YZ and XL performed transgenic, cell biological, and other functional analyses. SY and XF wrote the manuscript.

## Conflict of Interest Statement

The authors declare that the research was conducted in the absence of any commercial or financial relationships that could be construed as a potential conflict of interest.

## Footnotes

1 https://phytozome.jgi.doe.gov
