# Supplementary material for: Identification of ZOUPI Orthologs in Soybean Potentially Involved in Endosperm Breakdown and Embryogenic Development
Source: Front Plant Sci. 2017 Feb 8;8:139. doi: 10.3389/fpls.2017.00139 (PMC5296293; doi:10.3389/fpls.2017.00139)
Supplement: Supplementary file 1 [file Presentation_1.PDF]

Identification of ZOUPI orthologues in soybean potentially involved in endosperm breakdown and embryogenic development

Yaohua Zhang, Xin Li, Suxin Yang*, and Xianzhong Feng*

Key Laboratory of Soybean Molecular Design Breeding, Northeast Institute of Geography and Agroecology, Chinese Academy of Sciences, Changchun 130102, P. R. China

*Corresponding author:

Suxin Yang

E-mail: yangsuxin@iga.ac.cn

Tel. 0086-431-88542365

Fax 0086-431-85542298

OR

Xianzhong Feng

E-mail, fengxianzhong@iga.ac.cn

Tel. 0086-431-85655051

Fax 0086-431-85542298

Electronic Supplementary Material

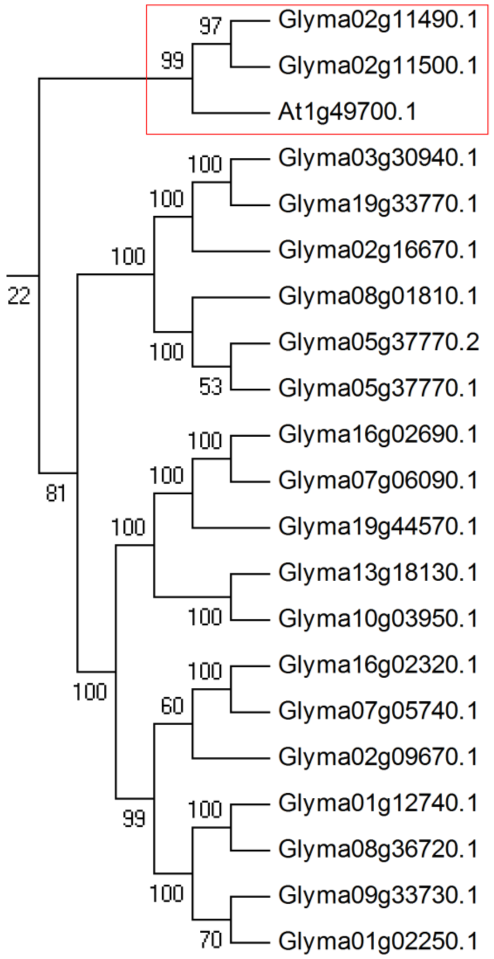

Supplementary Fig. S1 Phylogenetic analysis of the bHLH family proteins in soybean with AtZOU

The graph shows a part of this phylogenetic tree and the GmZOUs and AtZOU are in the same phylogenetic clade represented by red box.

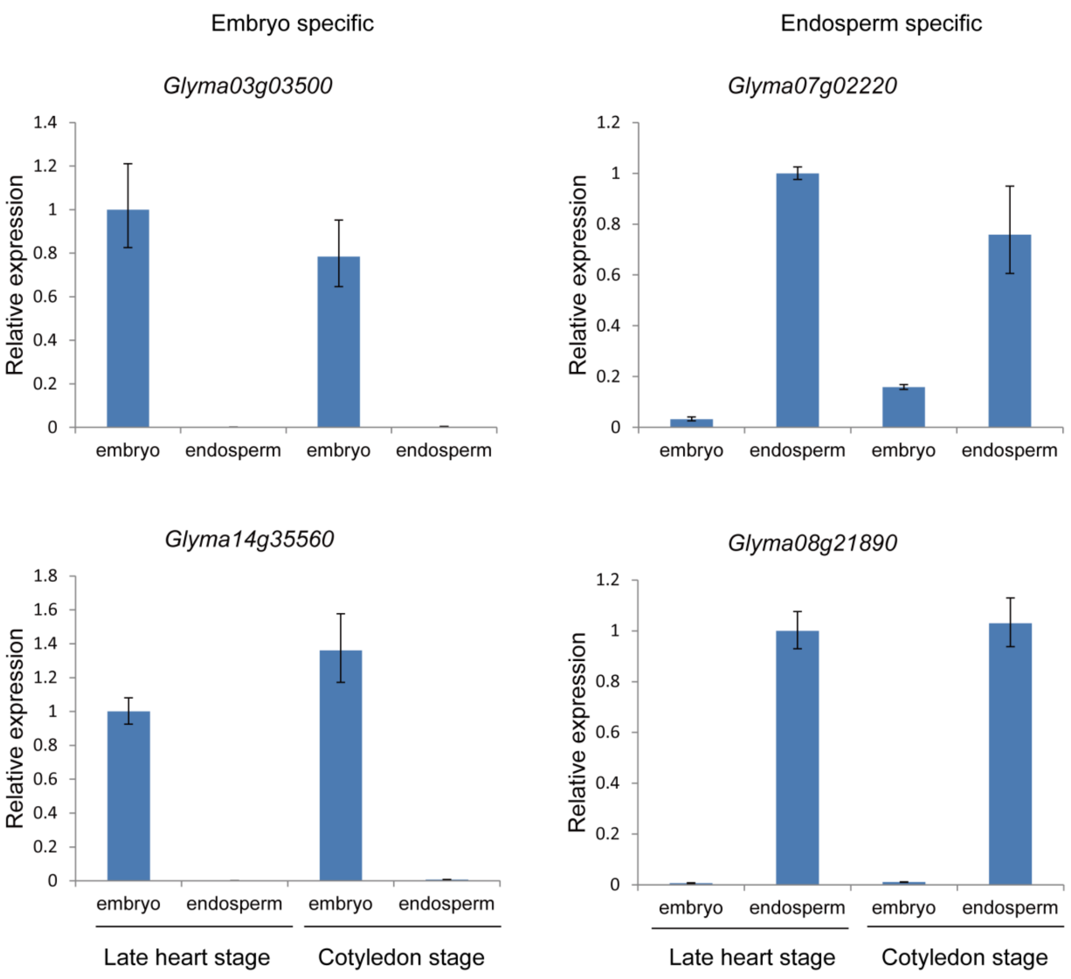

Supplementary Fig. S2 The expression of endosperm and embryo specific genes in dissected seed compartments detected by qPCR analysis

The embryo specific genes (*Glyma03g03500* and *Glyma14g35560*) and endosperm specific genes (*Glyma07g02220* and *Glyma08g21890*) were selected according to Danzer (2015). All of the genes showed tissue specific expression pattern as detected by qPCR. Primers used were listed in Table S1.

Table S1. Primers used in this paper

ID	Sequence	Note
OL0085	5'-CTCCCTCACTCTACCATGACCTTG-3'	GmZOU-1 gene clone (Forward)
OL0086	5'-GCCATAAAAGTCCGCTAATAGTTTC-3'	GmZOU-1 gene clone (Reverse)
OL0087	5'-CCACCTTGATGGCAGAAGATC-3'	GmZOU-2 gene clone (Forward)
OL0088	5'-CAGTTGCCTTTAGAACTGTCCTAG-3'	GmZOU-2 gene clone (Reverse)
OL1211	5'-TGGAACTACTTCAAATGCACTTTC-3'	GmZOU-1 Q-PCR (Forward)
OL1212	5'-TGAATTT GCATCTAGGAACTGATG-3'	GmZOU-1 Q-PCR (Reverse)
OL1213	5'-GGGAAGAAGAAATTAGAATTTGAC-3'	GmZOU-2 Q-PCR (Forward)
OL1214	5'-TCAGCTGAATTTTCAT CTGAAAC-3'	GmZOU-2 Q-PCR (Reverse)
OL1016	5'-CGGTGGTTCTATCTTGGCATC-3'	GmACT11 reference gene (Forward)
OL1017	5'-GTCTTTCGCTTCAATAACC CTA-3'	GmACT11 reference gene (Reverse)
ZOUSALIF	5'-GTCGACTGTGGTGGCATAATACGA-3'	AtZOU Promoter clone (Forward)
ZOUSALIR	5'-GTCGACTGCTCATTTTACCCTTTT-3'	AtZOU Promoter clone (Reverse)
OL2780	5'-GGTTGTTTGTGCAATATTGTCCAG-3'	ALE1 Q-PCR (Forward)
OL2781	5'-TCTAACGCCATAGCTTTGCTGTTG-3'	ALE1 Q-PCR (Reverse)
OL2586	5'-ACAGTGGTGAGGCCATTACAGATGC-3'	At3g06890 Q-PCR (Forward)
OL2601	5'-GAACGGAACAAGCACCACTAATG-3'	At3g06890 Q-PCR (Reverse)
OL2584	5'-AACCGCCTCAATACCCTCATCAAAG-3'	At5g03820 Q-PCR (Forward)
OL2653	5'-CCCTAAACTCTCAGCGGTAAAGTCTG-3'	At5g03820 Q-PCR (Reverse)
OL2582	5'-TCACAGCGACTTACGGTCAGAACAC-3'	At4g33600 Q-PCR (Forward)
OL2583	5'-TGATCTTGGTCCGGTTCTCATTAGC-3'	At4g33600 Q-PCR (Reverse)
OL2598	5'-TCTCTCGCAATCTTCGCTCTTCTC-3'	AtEIF4A1 reference gene (Forward)
OL2599	5'-GTTTCTGGTCAAACTGACGTGCATC-3'	AtEIF4A1 reference gene (Reverse)
OL3809	5'-TCGCCAGAGATCCAGACTACGACG-3'	AtZOUPI Q-PCR (Forward)
OL3795	5'-CAAGGGATTTGATTGAACTCACTGC-3'	AtZOUPI Q-PCR (Reverse)
OL6559	5'-TGCACCAGCTTGTTCAAGAATGTC-3'	Glyma03g03500 Q-PCR (Forward)
OL6560	5'-TGAAGGTGCATTACGAATCTCCTC-3'	Glyma03g03500 Q-PCR (Reverse)
OL6561	5'-CAAACCCATGTGGGAATCATCAAC-3'	Glyma14g35560 Q-PCR (Forward)
OL6562	5'-GCAGGAAGGGATTGATCAACAGTG-3'	Glyma14g35560 Q-PCR (Reverse)
OL6565	5'-GCGAGAGGACACTGTTGAGATTAG-3'	Glyma07g02220 Q-PCR (Forward)
OL6566	5'-CGTCATCCTCATGAAGACCTTCAC-3'	Glyma07g02220 Q-PCR (Reverse)
OL6567	5'-GCATGGTAACGGCTACCATAGATG-3'	Glyma08g21890 Q-PCR (Forward)
OL6568	5'-GTAGGAGACGTTGTCCTTGGTGAG-3'	Glyma08g21890 Q-PCR (Reverse)

Supplementary Dataset 1. 21 sequences used in Phylogenic analysis

>AT1G49770.1

MTNAQELGQEGFMWGISNSDDSGGGCKRIEKEPLPSHPSHPSPEIQTTTVKKGKKRT

KRNDKNHEEESPDHEIHIWTERERRKKMRDMFSKLHALLPQLPPKADKSTIVDEAVS

SIKSLEQTLQKLEMQKLEKLQYSSASTNTTPTTTFAYAPSSSSSPTALLTPISNHPIDAT

ATDSYPRAAFLADQVSSSSAAAANLPYPCNDPIVNFDTWSSRNVVLTICGNEAFFNLC

VPKHKPGVFTSVCYLFEKYNMEVLFANVSSNVFWSTYVIQAQVNPSCENQLLGNGL

GVVDVFKQVSQE

LVLYFSSL*

>cassava4.1_028789m

METPDAQLGQKRNRKGVAKDGETVNSVGGAESEHEVHILTERERRKKMRNMFTSL

HALLPQLPAKADKSTIVDEAIKYIKNLQETLQTLQHQSQEKLQGVTIVDSEPSVITSHT

EALESREAFMALQGSSKSFTMATNMPLSFPVSLPPSCFQTWFSPNVVMNMCGGDAQI

SVCSLKRPGLLTSIFYILEKHKLDVVSAHISSDQFRSIYMIHVHAGGVSGQYPEALSVE

DTFKLAAGEMNLWLLSC*

>cassava4.1_030210m

MEITAVAQVGQKRNRKGVAKNGETVKSGSAAESEHDVHILTERERRKKMRNMFSN

LHALLPELPAKADKSSIIDEAVKYIKILQETLQTLEKQKEEKLHGATIDESEPSVITSHK

EVFESREAFLAIQGPSKGSSMATNMPHSCPVSLSPSCFRTWFSPNVVMNISGNDAQIS

VCSLRRPGFLTSIIYILEKHKLDVVSAHISSDQFRSIYMIHVHAGWGSGQYPEALSVED

TFKLAAGEMNLWLLSC*

>cassava4.1_030216m

MKLSYGKTRHGFYPIPTILQLRNQKRRKELAKSKPSRGDTNKNDKGSDDSDGKHQK

GTGGGSGGGGGGESDHEIHIWTERERRKKMRNMFANLHALLPQLPPKADKSTIVDE

AISYIKTLQQTLQKLQKQKLERLQGVTTFSYEPTNISSVCPQKLTDQSRESFLADHGCF

NKLAIVSTKPNSNNVLPIERYPVLFQTWTSSNVVLNICGDEAQISICSSKRPGLFTTICY

ALEKHSIEMISAHVSSDCNQSMYMIHAHVNRASAEAIPVEETYKQAVGEIMCWVSS*

>Glyma.02G103100.1

MYHSPIFLLNPKTKPLSLSFFLFLFLSTLMAEDQGSHAIDFHLGNHLLVSSNSDNSGES

KNNMKPLNQQNEEGEALVNKKRNREIASENEKDNITSDEGLDGKHPESNNEMFDDL

RGLLPQLPSEADEATVIKVAANYIKGLEESRKRLGKITKRMFPYVFKEIMGTSDTILIN
GKKKLEFDKVWTSSNMVLNIHANWAQFTVCSAYKPHLLNTITSVLERHNIEVIAANV
SFNHNENGKTCMILVHAKQVSDENSAEETYKDASGEIFSLIS*

>Glyma.02G103200.1

MTLLTVEDQSLHDMNFLWENHSWDPSNSDNSGESKENMKSLDQVEEKDEALMNKK
RNRDQGNITSGEGKGEKCRESDHEMHIWTERERRKKMRNMFASLHALLPHLPSKAD
KSTVVDEAVAYIKNLEKTLEKLEKQKQERVQCVSTFGCEPSMFVTGQGSSNNNISNA
IIGTTSNALSFPVAFDKTWASANLVLNIFGDEAQFSICTAHKPGLMTTIGFVLEKYKIE
VITANISCIGYGNACMIQAHGKRVSHQFLDANSVEEIYKQAAGEIMLWIG*

>GRMZM2G147685_T01

MSQEGANLPQEVVGSHDQATAPHGSIPAPADSNPSSVSNLASAVNNGGSSECASPAV
LSAGEDNNAASSKTASPAVLSAGEDNNAASSKTASPAVLHAADDNNAASKIAGPAV
LLAGEENKAKLKLASPASLLAGEEKGNNADEFKLASPRMLHDCDDNNAGSHAGVR
NCNAAQSKLANPAVLHAGDDKKGGSKIAGPEVPHASVTSTTGSKLNGPAALHAGND
NDSGGSRLPNPAVLHASKDMDNNAGRPKFASPVLLHAGEDNKARTKLAIQAGGGG
NAGAGSSRLTRSAALHAGKDNGAGSKPAIQVVPRLHAGGKDNNAGSFKAARPSAAD
SGESNAKEGKSNVAGEQRAREAGVGCGGGKGNAAAVEDVDHDLHIFTERERRKKM
KNMFSTLHALLPQLPDKADKATIVGEAVTYIKTLEGTVQKLEKLKLERKRALAAQQ
QLMAGAGSNRASSARHPAPAPSSPSSSSREANVADMVHGWHAQQAAANKALAAEA
GAGGSSSAAASLPRGAVPFPAPAAGFQTWSGQNVVVSVASNEAYINLHSPRQPAGTL
TKALFVLERHRIDVVTTTISTQDGFHMYGIHARVNSASASARFPENLCAEDRFKLAVS
EMLQLINI*

>Medtr4g085660.1

MAMSVDHDQGVQTGFIWENHPWGVLPISDNLGESSKKRIDTKPLNQKEGINEGDAP
VRKKRSRGGVVIRSENNITTDEGEDRKYRDFDHEMHILTERERRKKMRNMFDSLHA
LLPELPSKADKSTIVDAAMKHIKNLEEIKEKLEKKKQEMLKSVSPLGSESSVINSQWH
PYESREAFLADQGSSSYNNNLSNAIVTSNPSNAFSISPPQQVGFQTWSSQNVVLNICG
GEAQFCICSTKKPGLLTTIALVLEKHKIDVISANIMCNANGNFYMIMAHAKQASHDA
NSMKETYKHAAGEIM

MWIA*

>Medtr4g085700.1

MAKSVDHDQGVQTGFIWENHLWGDLPNSDHNLGESSKKRVDTKPLNQNEGINEAE

APVSKKRNWGGVVIRSENNITTGEEKDKKYRDFDHEMHILTERERRKKMRNMFDSL

HALLPELPSKADKSTIVDAAVKHIKNLEEIKEKLEKKKQEMLKSVSPLGSESSVINSQ

WHPYESREAFLADQGSSSYNNNLSNAIVTSNPSNAFSISPPQQVGFQTWSSQNVVLNI

CGGEAQFCICSTKKPGLLTTIALVLEKHKIDVISANIMCNANGNFYMIMAHAKQASLG

ANSMEETYKQAALEI

MTRIA*

>Os02g34320.1

MAQEGTSSNAPPAAASMGSGDGDNKEGTGESGNNQLLLPAIAASADKGKGVVAGT

GNVDAKGKTTAMAPAASSTNAPNNQGGGGGGGGRSRERMHIFAERERRRKIKNMF

TDLRDLVPSLTNKADKATIVGEAISFIRSLEETVADLERRKRERNSLAARCARLGLGG

SSSSSAPPPPPPPAAADDTAAVMPPAPAVPPPDAAAVTAGPEPAPAPAPGTLMVWSGP

SVVLNLCGGDQAFINVSVARRPGVLTMIVDVLERHSIDVVTAQIASDLSRSLFTIHTSV

DRERGMFMDTATAEE

IYQLAVSEIM*

>Os04g35010.1

MSQEGADLSQDVDESPYHTAVVTTNNLVRSIKAEKSNSSSSSGKPVETDIGLKVASPT

MFGFNTKIEGTGKNMAVKREEGEGGGRPGVSSGVSTRDTNGKGKNAMDMEHALHI

WTERERRKKMKNMFSTLHGLLPKIPGKTDKASIVGEAIGYIKTLEDVVQKLETIKTER

VRAHQWAAAAAAAVAANGGGEGSSHSHSQPPRHATAVTVAVAEPAPVAAAVNAQ

APQKKAAAAAAPTLQTWSAPNITLTMAGVDAFINMCLPRQRASFTTVAFVLEKHQID

VVTSTISADHDKSLFSVHVRLNEASLQSTEGLTPEAKYKLAVSELMVRLAE*

>TA28041_3332

MEYCNRQESVTEPNLDHDQASAGCKPREPKSIDHKKSAQALKLGELGLGNSLMKSIE

PEDQGIHIWTERERRKKMRSMFSNLHSLLPHLPAKADKSTIVEEAISYIKTLQQSLHVL

ENQRLDKARAASTLEFELSSTFHEMQQQHHVSTATMRSASEAPAFYHPLTAECSASS

GFEPWISRQYSRTISTGSPAIDHCFFQTWSSPNVVLSVCGDDAHMMICSSPPKQGLLTT

IFYTLEKHKVDVVTAHISSDSCRSMYMIHAQAHAMVNGGDQFLESKASEEAFRSAA

GELVFYLSS

>TA18136_3352

MAQFGMNCNGSDYHPGEQDHEKYRLQSTNIAQDIDFLSNDIQDQYHMEYCKRQESV
TEPNLDHDQAPHGCCKSREPKSIDLKQSAQALNIRELGLGNSLMKSIGPEDQGIHIWT
ERERRKKMRNMFSNLHTLLPHLPAKADKSTIVEEAISYIKTLQQSLHVLENQRLDKA
RSATTLEFELSNTFHELQQQQHHVSAAAMRSSSETPGFYHPLIPECSTSSGFEPWISKQ
YSRTISAGSPAIDHCNFFQTWSSPNVVLSVCGDDAHMMICSSPPKQGLLTTIFYTLEK
HRVDVVTAHISSDSYRSMYMIHAQAHTMVNTGDQFLDSKSSEEAFRSAAGELVIYLS
S

>Potri.001G305100.1

MSEEGEHESFLWDNQTWDLSNSDNLGGSEEKIGKNIKLPGSSSNSQAEIGIKEQENKA
EKRGQMHKSNGKGSVGEVKEGKGGGESDHETHIWTERERRKKMRTMFSNLHALLP
QLPPKADKSTIVDEAMNYIKTLQHTLQKLQKEKLERLQGATTFGYEPSLIAPQMQAD
SREAFLADQVSSSNLAISTTKSLPSVSRYPVLFQTWTSSNVVLNICGDEAQISICSPKNP
GLFTTICYVLEKHNVEVLSAHVSSDCSRSMYMIQAHVSDHFLFVTTSFLFFWQFD*

>Potri.014G025900.1

MSTIKTCRETLLAAFTDNSDNYKKLVSKSYNNYSATSSLMDTEIPAVAVVPGQKRSR
KGGDARNKKVVNGGGSGESEHEIHIWTERERRKKMRNMFSSLHALLPQLPAKADKS
SIVDEAVKYIKTLQQTLQTLQKQKVEKFQGAIIDFEPSVITSLTDTVGSREASFAALGP
SKNSPLTSKMSQNSFSVSLSPACFQTWFSPNVVMNMCGDDAQFSLCSTRKPGLLATIL
YILEKHNLDVVSAHISSDQYRSIYMIHAHADGASDQYPEAMSVEDTFKLAAGEMNL
WVMSC*

>Pp1s371_38V6.1

MEPSRRPASLQSERFWSAVRMQSELMAVVTNVAITGESGYGAAPDNPHATITHPKTS
YDSDTRYRILSSRVHEHLNGCLADQLMDIQQGMGVEASTPQLHVEDMPQADRMSV
DNDAANLHAFANEFDTGWPTSGNESALVEDLHTRRLQLNGFQSFHNQIFNECAKINF
HSNSSPVSAQQLIMMCNQQKGVQNSRSGSWFSDRIEHPNTMCRPTSDNVFTNRDHAS
SSSQFDKMQPLGGKIGDNVTHPNCLPSVAQKQSQAPRLQTELVGCAPISAFYSAEPTC
FQSPPQMQSIHNAVSFSLGDEIGQTVPIGANASQENGCATTKQVPNQIDETGNCNARL
HSCEDQGSLDLQRVWCQPSQAITSFQRCGSATTSCGHASGLAPDRSKPGSTRTSEDG
GKSSPPVHRTPSGGKHRALTNPKKGRKQKLPGKTTTQAFLNKAVSQRESHIWSERQR

RRSMNQLYTTIRALLPHQSVKTDKATVVMDIINYIRAMQADLEVLSRRRDQLLAALN
LRRQPSQVFSAHGLTCVDHTSDASVLTAVTTLPPPGSVSCLTSFLGNNVAIHICGQHV
FVTITSAPQSRPGLLAQIISTLTNYNLDVLSATVNSRDNTTAYALSVETSQSVESLGDD
LHTQLQIVINNFSPSDAKEP*
>Sb04g022260.1
MAQNGPHGHQVDAAPPTTVAVFSLTSAGPNGNGGSSAGNMENLDASGMGKKNLIP
DLNMEERDDSAGGSKDKPRSEAKVEVAAADADADADADKGKSIAAAGDDDGEPNV
NIALERERRKRMKDLFRSLQDLMPHVPQKTDKATLVGEAITYIKVLEEKADMLGKQ
ALAARQAAARGEASSSSSLLSLRAMPPTAQGMAALCSWNAPRGWGGVPVQPAAPA
VPASTSPMRCKTWAGPNMVLTVANDNAYISVWAPRRANTLTMVMSVLDNHGIDVI
TAQISSDRVRALFMIYAHVTGIGGENRRSSEEVYQRAVSEIVYRLHNNNNNNNNNG
*
>Sb06g016740.1
MLHDYDNSKAGSHAGVDNNAARSEVASPAVLHAGDGNNGGSKIASPAVPQAIVENP
TGSKLDSPAVLHADEHNNAGFKVPNPAVLHASKDMDNNTERPKFGSSLLLHAGKDN
KARTKLAIQAMSHASGNAGSSRLTRSAAAALHAAKSNASNLAIQVAPRHHAGKDNI
AGSSKAARPAAVGAGAGESNAKEGKNNIARGQQRALEADVGGGSGKGNAAALEDT
DHDLHIFTERERRKKMKNMFSTLHALLPDLPDKADKATIVGEAVTYIKSLEGTVEKL
EKMKLQRKRALAAQQQLQQHGQQLLVGAGSGSSRASSARHPAPALPAPAAPAPAPA
ASSSREASLADMVHSLEVQQASVAAANRALAVAAAAAAAGGSGSGSSSGAAAANA
LSLPRGAVPFPAPATGFQTWSGQNVVVSVANNEAYINLHCPREPGTLTKVLFVLERH
SIEVVTTTISAHDGFRMYGIHARANPASASARFPQNLGAEDRFKLAVSEIVQLINI*
>Sb07g002210.1
MENHHSSAAPPNAKDGSSGIPEENKDVAVPNLSSAPEVAATVAASNDDDVSKGKNV
VAADNEELKPHIVSERSRRNRLRDYFGELKAYIPQIPEKSDKATIVEHAIDYIKYLEKM
KAMLEKRKQELALARQVGVVAAASSSSAPPPPPPPPPQTSHGMAVAAMPSDVPAGA
CSYVPPPPPPQPAVPVPAPQLLPATMSSDVVPQPQPLQPAPPQARIITATPVGFQTWSW
PDLVLSVSNDTAHISVSAPRHRGMWTKVMVLSVLNKYGIDVVTAQVDSDAVRSVFN
IYARNLKAKQAKVTAMGGGNPSALEVYQLAVSEILV*
>Smo413316

MPPRRRRSKSCSTATATATAAAPLPDGSIEQSIHQMLKSPLLEETRLPSFELEDFPEWW
PAAATSSDQGASPMATFESMDDPLLHFPQELPETHSTLQGICSPASSVLPCLTSPSPTQ
FVAPPALPPVGKAAGAGSKRSSRRTLAATHDLGVDTKSKSISPRESHILSERQRRKGM
NHLFSTLASLLPETCSKSDKSTIVSEIISYIHLLRKDLEDLDKKRSDVLRSASPRAAMAI
KDSGSPSPSICTTTNDRGSKNAGGGDDHPGMIQQQSQQASNVILSVCGSDAFITMICA
SKNRSVFSKVLLLLDHHKFRVLDANISTNASTTFHYFHVKALNSQLPKDALQRDLQS
LTASETKNETSSS*

>Thhalv10012185m

MTNAQELGQEGFVWGISSSDDSGGGCKKMEKQQPSLQLSHPLEISISMDKKIAKGKK
RTKRNDKNHVEESPDHEIHIWTERERRKKMRDMFSKLHALLPQLPPKADKSTIVDEA
VSSIKSLEQTLQNLQMKKFEKLQYSSASNTTPTTSNFPYDPSSSSSPTTLLTPVSSNHPQ
ILTLGTTAAESYSREAFLADQISSSSKNLPYPCNDPIAAFDIWSSRNVVLNICGNEAFFN
LCCPKVKPEVFTNVCYLFDKYNIEVLFANVSSNVFRSTYMIQAQVNPSYENQLLGDGI
GVGEIFKQAAQELVLYFSSS*
